# 基于多壁碳纳米管改进QuEChERS法结合气相色谱-串联质谱检测茶叶中10种拟除虫菊酯类农药残留

**DOI:** 10.3724/SP.J.1123.2021.11015

**Published:** 2022-05-08

**Authors:** Ruihan XU, Qianwen XIE, Xujun LI, Hongli ZHAO, Xuebin LIU, Yuanlong WEI, Aidong QIU

**Affiliations:** 1.成都大学食品与生物工程学院, 四川 成都 610106; 1. College of Food and Biological Engineering, Chengdu University, Chengdu 610106, China; 2.四川省中安检测有限公司, 四川 成都 610100; 2. Sichuan Safety Testing Center Co. Ltd., Chengdu 610100, China; 3.四川茂华食品有限公司博士后创新实践基地, 四川 眉山 620038; 3. Postdoctoral Research & Development Base, Sichuan Maohua Food Co. Ltd., Meishan 620038, China; 4.四川大学轻工科学与工程学院, 四川 成都 610065; 4. College of Biomass Science and Engineering, Sichuan University, Chengdu 610065, China; 5.榆林学院化学与化工学院, 陕西 榆林 719000; 5. School of Chemistry and Chemical Engineering, Yulin University, Yulin 719000, China

**Keywords:** 多壁碳纳米管, 气相色谱-串联质谱, 拟除虫菊酯类农药, 茶叶, 基质效应, multi-walled carbon nanotubes (MWCNTs), gas chromatography-tandem mass spectrometry (GC-MS/MS), pyrethroid pesticides, tea, matrix effect (ME)

## Abstract

利用多壁碳纳米管(MWCNTs)QuEChERS法提取茶叶中拟除虫菊酯类残留农药,采用气相色谱-串联质谱(GC-MS/MS)分析测定,建立了一种灵敏度高、可靠性强的茶叶中农药残留检测方法。比较了单壁碳纳米管(SWCNTs)、MWCNTS、氨基化多壁碳纳米管和石墨烯4种碳纳米材料和其不同用量下的净化效果;采用正交试验设计对前处理最佳实验条件进行筛选,并对实验影响因素进行方差分析。结果表明:提取溶剂、碳纳米材料种类对10种拟除虫菊酯类农药回收率的影响具有极显著统计学差异(*p*<0.001),提取时间对回收率的影响有统计学差异(*p*<0.05),碳纳米材料用量对回收率影响不显著(*p*>0.05);最佳样品前处理条件为以乙腈为提取溶剂,超声提取35 min,净化剂为60 mg MWCNTs、200 mg PSA和200 mg C18。方法学考察表明,10种拟除虫菊酯类农药在0.01~2 mg/L范围内线性良好;检出限(LOD)为0.001~0.01 mg/kg,定量限(LOQ)为0.005~0.04 mg/kg;绿茶样品空白基质加标试验中,10种农药的回收率为91.4%~109.7%,相对标准偏差为0.12%~9.80%(*n*=6)。对花茶、绿茶、红茶3种茶叶基质进行基质效应(ME)评价,结果发现净化剂中加入MWCNTs在绿茶和红茶基质中能有效降低ME。利用该方法检测了市售120份茶叶中拟除虫菊酯类农药的残留,多个样品中检出目标物,但均未超标。该方法检测灵敏度高,可靠性好,具有良好的回收率和稳定性,能满足茶叶中农药残留快速定量分析的要求。

## Abstract (en)

A modified QuEChERS method, based on multi-walled carbon nanotubes (MWCNTs), was established for the detection of 10 pyrethroid pesticides (cyfluthrin, flucythrinate, fenpropathrin, bifenthrin, cyhalothrin, permethrin, cypermethrin, etofenprox, fenvalerate, deltamethrin) in tea, in combination with gas chromatography-tandem mass spectrometry (GC-MS/MS). The purification effects and dosages of four carbon nanomaterials, viz. single-walled carbon nanotubes (SWCNTs), MWCNTs, amino-modified MWCNTs, and graphene, were compared. An orthogonal experimental design was used to determine the optimal experimental conditions for sample pretreatment. The experimental factors governing the process were analyzed using variance. The results showed that the optimized sample pretreatment parameters were as follows. Acetonitrile was used as the extraction solvent with ultrasonic extraction for 35 min, while 60 mg MWCNTs, 200 mg PSA, and 200 mg C18, were used as purifiers. The effects of the extraction solvent and the carbon nanomaterials used on the recoveries of the 10 pyrethroid pesticides were significantly different (*p*<0.001), and the effect of extraction time on the recoveries was statistically different (*p*<0.05). The dosage of carbon nanomaterials had no significant effect on the recoveries (*p*>0.05). Good linearities were observed for the 10 pyrethroid pesticides in the concentration range of 0.01-2 mg/L. The limits of detection (LODs) and limits of quantification (LOQs) were in the ranges of 0.001-0.01 mg/kg and 0.005-0.04 mg/kg, respectively. The average recoveries of the pyrethroid pesticides spiked into blank samples of green tea were 91.4%-109.7%, and the relative standard deviations were 0.12%-9.80% (*n*=6). Furthermore, the matrix effects (MEs) of scented green tea, green tea, and black tea were evaluated. It was found that the addition of MWCNTs to the purifier can effectively reduce the matrix effect in green tea and black tea matrices. The developed method and the national standard method were used to detect the residues of the 10 pyrethroid pesticides in 120 tea samples available in the market. The results showed that cyfluthrin, deltamethrin, fenvalerate, permethrin, fenpropathrin, cypermethrin, bifenthrin and cyhalothrin were detected, and the contents obtained with the two methods were similar. Although pyrethroids were detected in most tea samples, the contents of all pesticide residues were below the maximum residue limits (MRLs). Therefore, the developed method is suitable for the rapid quantitative analysis of pesticide residues in tea.

茶叶最早起源于中国,是我国重要的经济作物之一,拥有可观的市场价值^[1]^。茶叶中含有茶多酚、茶多糖、生物碱等物质,具有抗氧化、降血糖、抗癌和抗炎等保健功效^[2,3]^,也因其特殊的风味和口感,成为世界上消费最多的饮料之一^[4,5]^。茶树多生长在湿热环境中,利于害虫的繁殖,为防病治病,茶农多喷洒相关农药^[4]^,拟除虫菊酯类农药就是广泛使用的茶叶农药之一^[6]^。随着人们生活水平的不断提高,健康意识的不断增强,茶叶中农药残留的潜在危害引起人们的高度关注。由于茶叶中色素等物质含量较高,检测时容易产生基质效应(ME),给目标物测定带来严重干扰^[7]^。为减少ME对检测的干扰,目前常用的方法有:基质多重净化法、基质匹配校准方法、进样技术优化、内标校正法和同位素内标校正法以及分析保护剂加入法等。为确保检测方法能获得更准确的结果,需要对ME进行评价^[8,9]^。

近些年来,碳纳米材料因其独特的结构和优良的吸附性能^[10,11]^,在食品安全检测领域受到广泛关注,应用于检测前的样品处理净化过程。吴静娜等^[11]^利用MWCNTs磁性固相萃取-气相色谱-质谱法测定茶叶中8种农药残留,LOD为0.004~0.010 mg/kg;崔丽丽等^[12]^利用MWCNTs改进QuEChERS-气相色谱-质谱法检测黄芪中16种农药,在0.01~5.0 mg/L范围内,呈现出良好的线性关系;陈彬等^[13]^也采用该方法对韭菜中32种农药残留进行检测,在0.01~2 mg/L内线性良好,LOD为0.003~0.02 mg/kg;陈啟荣等^[14]^采用MWCNTs固相萃取净化-气相色谱-质谱联用测定茶叶中26种农药,在0.04~1.6 mg/L内具有良好线性,LOD为0.005~0.05 mg/kg。石墨烯作为碳纳米材料中的一员,比表面积大、电导率高,多用于电极材料、传感器等^[15]^,因其片层结构间易团聚,运用其作为吸附材料的研究较少,多用氧化状态的石墨烯作净化材料,如侯秀丹等^[16]^采用氧化石墨烯气凝胶固相萃取柱检测苹果中的有机磷农药,线性拟合良好,LOD为0.0002~0.0005 mg/L,获得了较好的检测效果。

国家标准GB 2763-2021《食品安全国家标准 食品中农药最大残留限量》对茶叶中10种拟除虫菊酯类农药明确规定了最大残留限量(MRLs)。针对越来越严苛的茶叶中农药MRLs,为解决茶叶检测中基质干扰的难题,本研究将单壁碳纳米管(SWCNTs)、MWCNTs、石墨烯等碳纳米材料作为优化QuEChERS前处理法中的净化剂,用于茶叶样品的基质净化过程,探讨茶叶检测中ME的影响,以期建立一种灵敏度高、可靠性强的茶叶农药残留检测方法,并将新建立的方法应用于市售茶叶中拟除虫菊酯类农药残留的检测。

## 1 实验部分

### 1.1 仪器、试剂与材料

Agilent 7890B GC system-7000D GC/TQ气相色谱-串联质谱仪(带反吹装置); HP-5MS UI (15 m×0.25 mm×0.25 μm)色谱柱(美国Agilent公司); XW-80A涡旋仪(海门市其林贝尔仪器制造有限公司); UPR-Ⅱ-10T超纯水器(四川优普超纯科技有限公司); TD-5M低速离心机(四川省蜀科仪器有限公司);电子天平(瑞士Mettler Toledo); BJ-300多功能粉碎机(德清拜杰电器有限公司); KQ-500DE数控超声波清洗器(昆山市超声仪器有限公司); MTN-5800水浴手动圆形氮吹仪(天津奥特赛恩斯仪器有限公司)。

10种拟除虫菊酯类农药标准物质:氟氯氰菊酯(cyfluthrin)、氯菊酯(permethrin)、氰戊菊酯(fenvalerate)购自Laboratory of the Government Chemist;甲氰菊酯(fenpropathrin)、氯氟氰菊酯(cyhalothrin)购自CATO Research Chemicals Inc.;氯氰菊酯(cypermethrin)、联苯菊酯(bifenthrin)购自坛墨质检科技股份有限公司;氟氰戊菊酯(flucythrinate)购自Chem Service Inc.;溴氰菊酯(deltamethrin)购自中国计量科学研究院;100 mg/L醚菊酯(etofenprox)标准溶液购自北京曼哈格生物科技有限公司。

乙腈、丙酮、乙酸乙酯、甲醇、正己烷(色谱纯)购自美国MORELLK公司;甲苯(色谱纯)购自成都市科隆化学品有限公司;氯化钠(分析纯)购自天津市科密欧化学试剂有限公司;*N*-丙基乙二胺(PSA)、十八烷基键合硅胶(C18)购自青云实验耗材有限公司;SWCNTs、MWCNTs、石墨烯(中国科学院成都有机化学有限公司);氨基化多壁碳纳米管(南京先丰纳米科技有限公司); 0.22 μm有机滤膜(天津市津腾实验设备有限公司)。

### 1.2 标准溶液的配制

单一标准溶液:分别准确称取氟氯氰菊酯、氟氰戊菊酯、氯氰菊酯、甲氰菊酯、联苯菊酯、氯氟氰菊酯、氯菊酯、氰戊菊酯、溴氰菊酯标准品,用乙腈溶解,配制成质量浓度为1000 mg/L的标准储备液。-18 ℃冻存,有效期约为6个月。

混合标准储备液:分别准确量取氟氯氰菊酯、氟氰戊菊酯、氯氰菊酯、甲氰菊酯、联苯菊酯、氯氟氰菊酯、氯菊酯、氰戊菊酯、溴氰菊酯标准储备液和醚菊酯标准溶液,用乙腈溶解,配制成质量浓度为10 mg/L的混合标准储备液,-18 ℃冻存,有效期约为1个月。

混合标准工作液:取一定量的混合标准储备液,乙腈稀释,现配现用。

### 1.3 实验条件

#### 1.3.1 加标方法

称取不含目标农药的空白绿茶,加入1.2节中配制的混合拟除虫菊酯类农药标准溶液后作为空白加标样品,空白加标样过夜,方法优化的单因素实验及正交试验设计所采用的加标样中农药的含量为0.1 mg/kg。

#### 1.3.2 样品提取

称取茶叶2.0 g(±0.01 g)于50 mL具塞离心管中,加入12 mL乙腈提取,涡旋混匀1 min,超声35 min;加入6 g NaCl,涡旋1 min;放入-18 ℃冷冻30 min, 4000 g离心5 min;取上清液,氮吹浓缩至2 mL,转移至含有60 mg MWCNTs、200 mg PSA和200 mg C18净化剂的15 mL离心管中,涡旋1 min, 4000 g离心3 min,取1 mL过0.22 μm有机滤膜,进小瓶,于-18 ℃保存备用。

#### 1.3.3 GC-MS/MS分析

色谱条件 2支Agilent HP-5MS UI色谱柱(15 m×0.25 mm×0.25 μm)串联;进样量:1 μL;载气:氮气,纯度≥99.999%;进样方式:不分流模式;色谱柱1流量:1.2 mL/min,色谱柱2流量:1.4 mL/min;进样口温度:250 ℃。柱温:初始温度100 ℃,保持1 min;以30 ℃/min升到130 ℃,不保持;再以20 ℃/min升到250 ℃,不保持;最后以10 ℃/min升到300 ℃,保持4 min;后运行2 min。

质谱条件 电子轰击(EI)离子源;离子源温度280 ℃;电离能量70 eV;碰撞辅助气为氦气;溶剂延迟4 min;采集模式为多反应监测(MRM)模式。具体参数见表1。

**表1:** 10种拟除虫菊酯类农药的GC-MS/MS分析参数

Compound	Quantitative ion pair (*m/z*)	CE/ eV	Qualitative ion pairs (*m/z*)	CEs/ eV
Cyfluthrin	198.9>170.1	25	162.9>127.0; 162.9>90.9	5; 15
Flucythrinate	156.9>107.1	15	156.9>77.0; 198.9>157.0	35; 10
Fenpropathrin	181.1>152.1	25	264.9>210.0; 207.9>181.0	10; 5
Bifenthrin	181.2>165.2	25	181.2>166.2; 166.2>165.2	10; 20
Cyhalothrin	197.0>141.0	10	208.0>181.0; 197.0>161.0	5; 5
Permethrin	183.0>77.1	35	184.0>169.1; 183.1>168.0	15; 20
Cypermethrin	163.0>91.0	10	163.0>127.0; 164.9>91.0	5; 10
Etofenprox	163.0>107.1	20	183.1>168.0; 376.0>163.1	5; 25
Fenvalerate	167.0>125.1	5	224.9>119.0; 208.9>141.1	15; 15
Deltamethrin	252.9>93.0	15	252.9>174.0; 250.7>172.0	5; 5

CE: collision energy.

### 1.4 基质效应评价

ME=(*A*_M_-*A*_S_)/*A*_S_×100%^[17,18]^,其中*A*_M_为待测物在样品基质中的峰面积;*A*_S_为待测物在纯溶剂中的峰面积。评价标准^[19,20]^: a. ME>0,基质增强;ME<0,基质抑制;b. ME绝对值在0~25%之间时,基质效应不显著;c. ME绝对值在25%~50%之间时,基质效应略显著;d. ME绝对值大于50%时,基质效应显著。

## 2 结果与讨论

### 2.1 不同提取溶剂对回收率的影响

分别在0.1 mg/kg的空白加标绿茶样品中加入乙腈、正己烷、丙酮、甲醇、乙酸乙酯5种有机溶剂进行提取效率比较,各目标农药的回收率结果见图1。结果表明10种拟除虫菊酯类农药在甲醇、正己烷、丙酮、乙酸乙酯、乙腈中提取的回收率范围分别为75.4%~133.8%、56.0%~132.3%、89.9%~134.6%、100.3%~150.1%、89.0%~104.8%。其中,正己烷提取的氯氟氰菊酯和氰戊菊酯回收率较低,甲醇提取某些菊酯类回收率偏低且目标农药间回收率差异较大。故本研究最后选择丙酮、乙酸乙酯、乙腈3种溶剂进行正交试验。

**图1:**
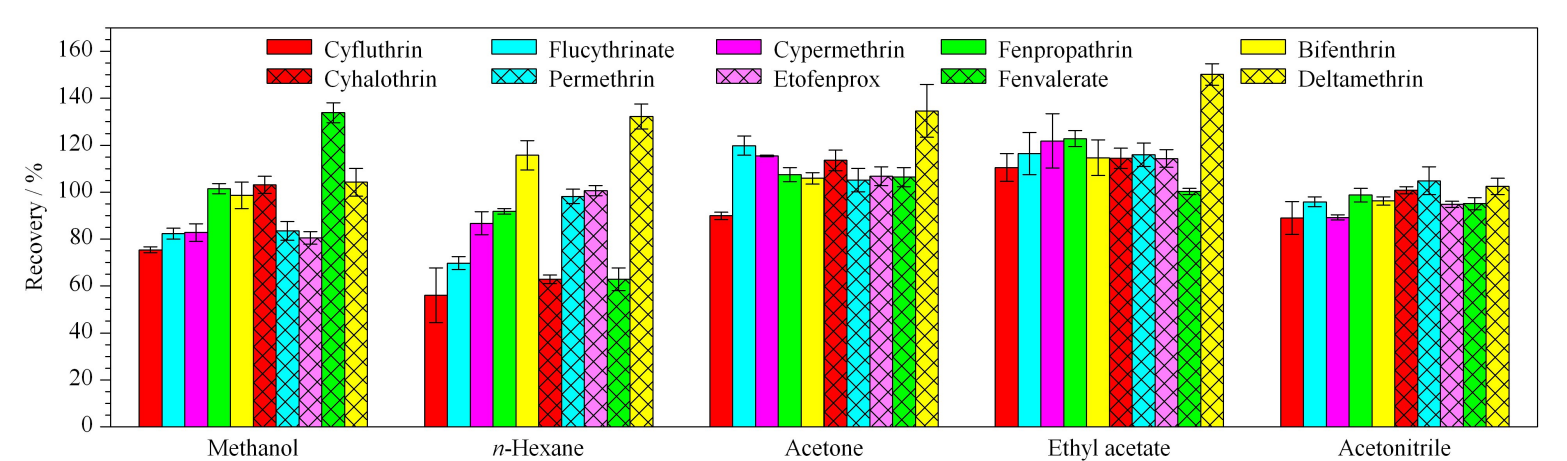
提取溶剂对各目标农药回收率的影响(*n*=3)

### 2.2 不同提取时间对回收率的影响

分别在0.1 mg/kg的空白加标绿茶样品中加入12 mL乙腈,涡旋1 min, 40 kHz超声辅助提取5、20、35、50、65 min。各目标农药的回收率结果见图2。结果表明,10种拟除虫菊酯超声提取5、20、35、50、65 min时平均回收率分别为54.9%、76.4%、87.1%、87.3%、86.3%。提取时间5~35 min时,各目标物的回收率随不同提取时间的增长而呈上升趋势,在35~65 min时,各回收率趋于平稳。综合考虑10种拟除虫菊酯类农药总体的回收率情况,选择超声提取时间20、35、50 min进行正交试验。

**图2:**
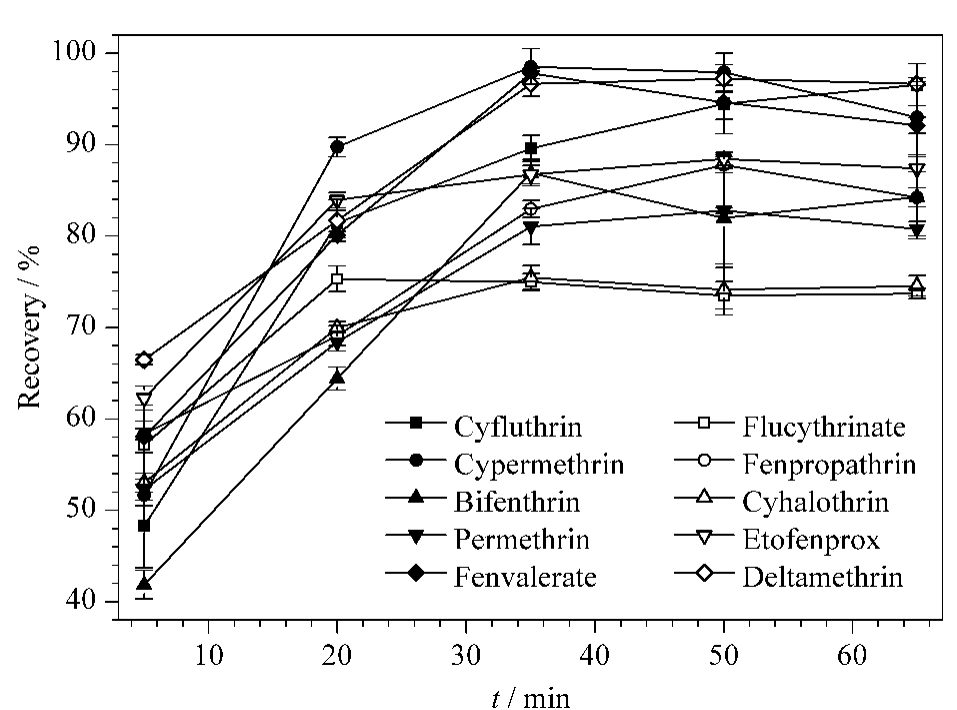
超声提取时间对各目标农药回收率的影响(*n*=3)

### 2.3 不同碳纳米材料及其用量对回收率的影响

在基础净化剂200 mg PSA和200 mg C18的基础上,分别加入0、30、60、90、120 mg 4种不同的碳纳米材料,考察其净化效果,目标农药的回收率结果见图3。

**图3:**
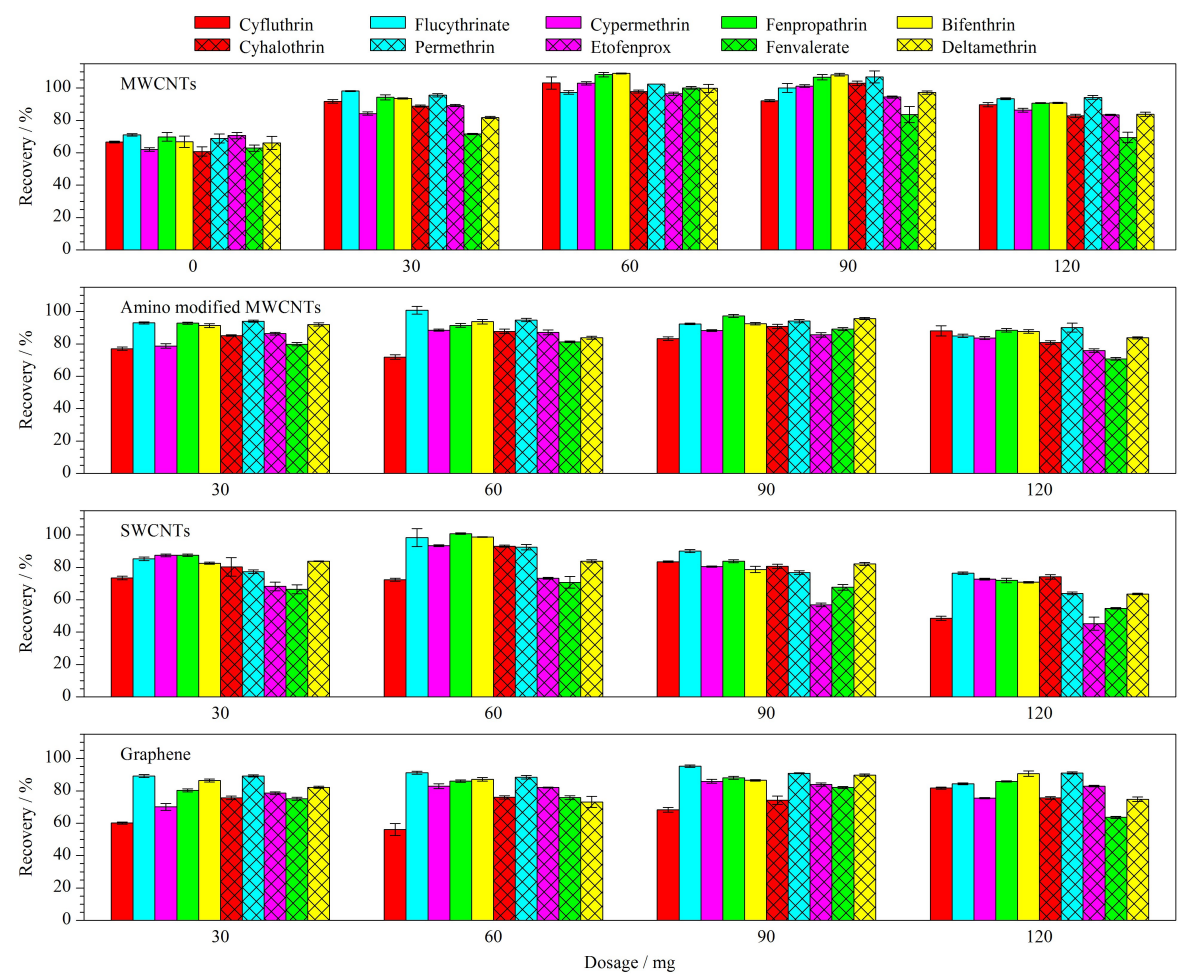
4种碳纳米材料及其用量对各目标农药回收率的影响(*n*=3)

在用量分别为30、60、90、120 mg时,SWCNTs净化组平均回收率分别为79.2%、87.7%、78.0%、64.2%; MWCNTs净化组分别为88.9%、101.7%、99.3%、86.4%;氨基化多壁碳纳米管净化组分别为87.0%、88.1%、90.9%、83.4%;石墨烯净化组分别为78.7%、79.9%、84.5%、80.6%。对4种碳纳米材料净化后各目标农药回收率进行比较,回收率较好的依次是MWCNTs、氨基化多壁碳纳米管、石墨烯、SWCNTs(见图3)。可见,在SWCNTs的用量为60 mg时回收率87.7%为最佳;石墨烯用量为90 mg时回收率84.5%为最佳,因石墨烯片层间易团聚使吸附净化效果受到影响而需要增加其用量,同时,因质量轻在实验中石墨烯的称量较难操作。综合考虑后,选择SWCNTs、MWCNTs、氨基化多壁碳纳米管3种碳纳米材料作为净化剂,同时选择碳纳米材料用量为30、60、90 mg进行正交试验。

### 2.4 正交试验设计及方差分析

在QuEChERS法采用200 mg PSA和200 mg C18基础净化剂的基础上,根据单因素实验结果,采用空白绿茶样品进行L_9_(3^4^)正交试验设计,并采用绿茶基质标准曲线定量。正交试验因素水平见表2,实验结果及极差分析见表3,方差分析见表4。

**表2:** QuEChERS法正交试验设计因素水平表

Level	Factors			
	A (solvent)	B (ultrasonic time/min)	C (carbon nanomaterials)	D (dosage of carbon nanomaterials/mg)
1	acetonitrile	20	SWCNTs	30
2	acetone	35	MWCNTs	60
3	ethyl acetate	50	amino modified	90
			MWCNTs	

**表3:** QuEChERS法L_9_(3^4^)正交试验设计实验结果及极差分析结果

No.	Columns						Average recovery/%	
	A	B	C		D			
1	1	1	1		1		89.5	
2	1	2	2		2		99.0	
3	1	3	3		3		89.0	
4	2	1	2		3		85.3	
5	2	2	3		1		81.4	
6	2	3	1		2		80.9	
7	3	1	3		2		84.3	
8	3	2	1		3		84.7	
9	3	3	2		1		85.6	
*K*_1_	277.5	259.1	255.1		256.5			
*K*_2_	247.6	265.1	269.9		264.2			
*K*_3_	254.5	255.5	254.6		259.0			
*k*_1_	92.5	86.4	85.0		85.5			
*k*_2_	82.5	88.4	90.0		88.1			
*k*_3_	84.8	85.2	84.9		86.3			
*R*	10.0	3.2	5.1		2.6			
Order		A>C>B>D						
Optimal level	A_1_	B_2_		C_2_		D_2_		

**表4:** QuEChERS法多因素方差分析结果

Source	Type Ⅲ sum of squares	df	Mean square	*F*	Sig.
A	491.53	2	245.76	49.30	0.000^***^
B	46.79	2	23.40	4.69	0.023^*^
C	150.03	2	75.02	15.05	0.000^***^
D	31.26	2	15.63	3.14	0.068
Error	89.73	18	4.99		
Total	809.35	26			

*: *p*<0.05 implies a statistical difference.

***: *p*<0.001 implies a very significant statistical difference

结果表明,QuEChERS法影响因素依次为A(提取溶剂)、C(碳纳米材料)、B(提取时间)和D(碳纳米材料用量)(见表3); A(提取溶剂)在1水平较其他2个水平回收率高,B(提取时间)在2水平上较其他2个水平的回收率高,C(不同碳纳米材料)在2水平上较其他2个水平的回收率高,D(碳纳米材料用量)在2水平上较其他2个水平回收率高(见表3);方差分析结果可以看出,A(提取溶剂)、C(碳纳米材料)对回收率的影响具有极显著统计学差异(*p*<0.001), B(提取时间)对回收率的影响有统计学差异(*p*<0.05), D(碳纳米材料用量)对回收率影响不显著(*p*>0.05)(见表4)。

依正交试验结果,筛选出最佳试验条件为:乙腈为提取溶剂;超声提取时间35 min; 60 mg MWCNTs、200 mg PSA和200 mg C18为净化剂。

### 2.5 基质效应评价

选取绿茶、红茶、花茶3种茶叶基质,对10种拟除虫菊酯农药在不同茶叶中的ME进行评价,结果见图4。

**图4:**
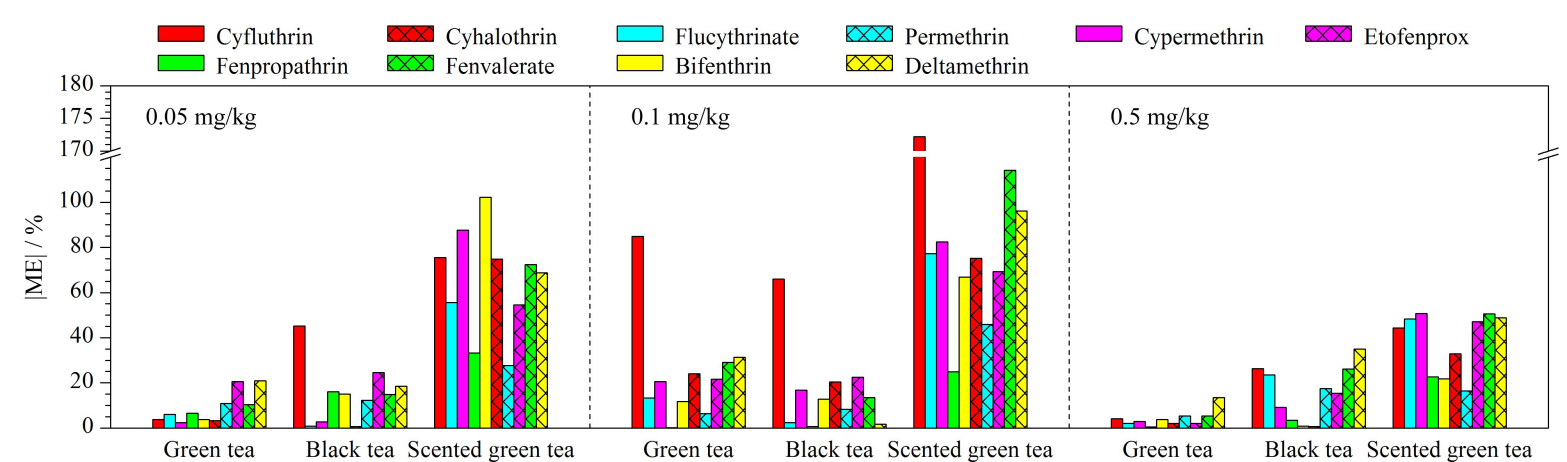
不同含量的目标农药在绿茶、红茶、花茶3种茶叶基质中的基质效应

在0.05 mg/kg水平下,各目标农药的ME绝对值在绿茶、红茶、花茶中分别为2.37%~20.85%、0.61%~45.10%、27.64%~102.18%;在0.1 mg/kg水平下,绿茶、红茶、花茶中分别为0.19%~84.88%、0.65%~65.91%、24.99%~172.20%;在0.5 mg/kg水平下,在绿茶、红茶、花茶中分别为0.47%~13.43%、0.66%~34.88%、16.43%~50.77%。即,各目标农药在绿茶中ME绝对值范围为0.19%~84.88%,在红茶中为0.61%~65.91%,在花茶中为16.43%~172.20%。可见,本方法下10种拟除虫菊酯类农药在红茶中受ME干扰较小,在绿茶中次之,在花茶中受ME干扰较大。

### 2.6 方法学考察

用空白绿茶样品基质配制一系列质量浓度为0.005、0.01、0.02、0.05、0.2、0.5、1、2 mg/L的混合标准溶液,对线性关系和相关系数(*r*^2^)进行考察。以3倍信噪比(*S/N*≥3)对应的含量作为LOD,以*S/N*≥10对应的含量作为LOQ^[21]^,结果见表5。

**表5:** 绿茶中各目标农药的线性范围、相关系数、检出限和定量限

Compound	Linear equation	*r*^2^	Linear range/ (mg/L)	LOD/ (mg/kg)	LOQ/ (mg/kg)	GB 23200.113-2018 LOQ/(mg/kg)^[22]^	
Cyfluthrin	*y*=2.9838×10^5^*x*-6.3133×10^2^	0.9998	0.01-2	0.01	0.03	0.05	
Flucythrinate	*y*=1.5012×10^6^*x*-2.2586×10^4^	0.9997	0.01-2	0.005	0.01	0.05	
Fenpropathrin	*y*=3.0136×10^5^*x*+2.1241×10^3^	0.9994	0.01-2	0.002	0.005	0.05	
Bifenthrin	*y*=2.7954×10^6^*x*+1.4523×10^4^	0.9994	0.01-2	0.001	0.005	0.05	
Cyhalothrin	*y*=2.9891×10^6^*x*-1.5338×10^4^	0.9998	0.01-2	0.002	0.005	0.05	
Permethrin	*y*=7.6320×10^5^*x*+2.7869×10^4^	0.9989	0.01-2	0.005	0.01	0.05	
Cypermethrin	*y*=2.5306×10^5^*x*-1.1162×10^3^	0.9999	0.01-2	0.01	0.03	0.05	
Etofenprox	*y*=3.0639×10^6^*x*-2.1589×10^4^	0.9999	0.01-2	0.005	0.01	-	
Fenvalerate	*y*=1.2430×10^6^*x*+3.7634×10^3^	0.9996	0.01-2	0.005	0.01	0.01	
Deltamethrin	*y*=1.9471×10^5^*x*-3.9377×10^3^	0.9994	0.01-2	0.01	0.04	0.05	

*y*: peak area; *x*: mass concentration, mg/L; -: not given.

用空白绿茶样品进行3水平6平行的加标回收试验,并计算方法的相对标准偏差(RSD),结果见表6。

**表6:** 绿茶中各目标农药在3个加标水平下的平均回收率和相对标准偏差(*n*=6)

Compound	LOQ^*^			2LOQ			10LOQ				
	Recovery/%	RSD/%		Recovery/%	RSD/%		Recovery/%		RSD/%		
Cyfluthrin	108.5	1.72		106.9	5.63		106.4				2.32
Flucythrinate	107.0	0.76		101.5	0.80		102.3				7.10
Fenpropathrin	98.5	9.80		108.7	5.11		109.2				1.09
Bifenthrin	102.0	2.53		100.7	6.30		100.3				6.66
Cyhalothrin	97.5	1.14		109.2	1.25		105.2				4.23
Permethrin	106.0	6.85		102.3	3.10		106.4				9.77
Cypermethrin	107.0	8.80		99.5	3.04		102.3				6.29
Etofenprox	104.7	6.82		107.3	8.07		100.8				4.21
Fenvalerate	102.6	0.12		109.7	6.21		109.3				3.69
Deltamethrin	105.8	6.03		106.4	6.73		91.4				5.54

* The LOQ for deltamethrin is 0.04 mg/kg, the LOQ for cyfluthrin and cypermethrin is 0.03 mg/kg, the LOQ for flucythrinate, permethrin, etofenprox and fenvalerate is 0.01 mg/kg, and the LOQ for the remaining three compounds is 0.005 mg/kg.

实验结果表明,基质混合标准曲线在0.01~2 mg/L范围内,*r*^2^为0.9989~0.9999,线性良好。本方法中目标菊酯类农药的LOD为0.001~0.01 mg/kg, LOQ在0.005~0.04 mg/kg之间,在GB 23200.113-2018中相应菊酯类农药的LOQ范围为0.01~0.05 mg/kg(见表5)。此外,其他文献采用MWCNTs净化茶叶基质的方法中LOD为0.004~0.01 mg/kg^[11]^和0.005~0.05 mg/kg^[14]^,综合比较发现,本方法检测灵敏度较高。

10种拟除虫菊酯类农药在3个加标水平的回收率范围为91.4%~109.7%,RSD为0.12%~9.80%(见表6),说明该方法具有良好的准确性和精密度,可以满足茶叶中拟除虫菊酯类农药的快速定量分析。

### 2.7 实际样品检测

分别采用本方法和国家标准方法GB 23200.113-2018中QuEChERS方法^[22]^对样品进行检测。对120份市售茶叶样品,分别采用相应的红茶、绿茶、花茶3个基质标准曲线进行定量分析。

结果表明,120份市售茶叶中氟氰戊菊酯和醚菊酯经2种方法检测均无检出,氟氯氰菊酯、溴氰菊酯、氰戊菊酯、氯菊酯、甲氰菊酯、氯氰菊酯、联苯菊酯、氯氟氰菊酯经国标方法检测的检出率分别为1.67%、1.67%、3.33%、5.83%、26.67%、32.50%、55.00%、60.83%;经本方法检测的检出率分别为1.67%、4.17%、5.00%、6.67%、31.67%、44.17%、60.83%、65.00%,两种方法检测结果相近。虽然目标农药有检出,但所有样品的农药残留量均未超标,残留量均低于GB 2763-2021中规定的MRLs值。本方法所用的净化材料MWCNTs净化效果较好,降低了检测干扰。

## 3 结论

本文通过对SWCNTs、MWCNTs、氨基化多壁碳纳米管和石墨烯4种碳纳米材料进行前处理方法的筛选优化,建立了一种基于MWCNTs优化的QuEChERS法结合GC-MS/MS检测技术,用于茶叶中拟除虫菊酯类农药残留的检测。该方法能够满足茶叶中农药残留安全检测等相关要求。茶叶空白基质加标试验表明其回收率和精密度均满足SN/T 5326.2-2020和GB/T 27404-2008的要求。相较于传统的QuEChERS方法,本方法灵敏度高,可靠性好,可作为茶叶中农药残留快速定量分析的一种新方法。
